# COVID vaccination and post-infection cancer signals: Evaluating patterns and potential biological mechanisms

**DOI:** 10.18632/oncotarget.28824

**Published:** 2026-01-03

**Authors:** Charlotte Kuperwasser, Wafik S. El-Deiry

**Affiliations:** ^1^Department of Developmental, Molecular and Chemical Biology, Tufts University School of Medicine, Boston, MA 02111, USA; ^2^Laboratory for the Convergence of Biomedical, Physical, and Engineering Sciences, Tufts University School of Medicine, Boston, MA 02111, USA; ^3^Laboratory of Translational Oncology and Experimental Cancer Therapeutics, Department of Pathology and Laboratory Medicine, The Warren Alpert Medical School of Brown University, Providence, RI 029121, USA; ^4^Hematology-Oncology Division, Department of Medicine, Brown University Health and The Warren Alpert Medical School of Brown University, Providence, RI 029121, USA; ^5^Legorreta Cancer Center at Brown University, The Warren Alpert Medical School of Brown University, Providence, RI 029121, USA

**Keywords:** COVID, vaccine, cancer, infection, lymphoma, leukemia, sarcoma, carcinoma

## Abstract

A growing number of peer-reviewed publications have reported diverse cancer types appearing in temporal association with COVID-19 vaccination or infection. To characterize the nature and scope of these reports, a systematic literature search from January 2020 to October 2025 was conducted based on specified eligibility criteria. A total of 69 publications met inclusion criteria: 66 article-level reports describing 333 patients across 27 countries, 2 retrospective population-level investigations (Italy: ~300,000 cohort, and Korea: ~8.4 million cohort) quantified cancer incidence and mortality trends among vaccinated populations, and one longitudinal analysis of ~1.3 million US miliary service members spanning the pre-pandemic through post-pandemic periods. Most of the studies documented hematologic malignancies (non-Hodgkin’s lymphomas, cutaneous lymphomas, leukemias), solid tumors (breast, lung, melanoma, sarcoma, pancreatic cancer, and glioblastoma), and virus-associated cancers (Kaposi and Merkel cell carcinoma). Across reports, several recurrent themes emerged: (1) unusually rapid progression, recurrence, or reactivation of preexisting indolent or controlled disease, (2) atypical or localized histopathologic findings, including involvement of vaccine injection sites or regional lymph nodes, and (3) proposed immunologic links between acute infection or vaccination and tumor dormancy, immune escape, or microenvironmental shifts. The predominance of case-level observations and early population-level data demonstrates an early phase of potential safety-signal detection. These findings underscore the need for rigorous epidemiologic, longitudinal, clinical, histopathological, forensic, and mechanistic studies to assess whether and under what conditions COVID-19 vaccination or infection may be linked with cancer.

## INTRODUCTION

The COVID-19 pandemic and the widespread deployment of novel mRNA- and viral-vector based vaccines have reshaped the landscape of human immunology [1–4]. Never has such a large proportion of the global population been exposed simultaneously to nucleic acid–based immunogens, lipid nanoparticle (LNP) delivery systems, and repeated booster regimens over a relatively short period. The unprecedented scale that was marshaled in response to the COVID-19 pandemic has generated and continues to generate extensive clinical, molecular, and epidemiologic data, revealing biological responses that extend beyond traditional vaccine-induced immune activation and responses. These include a spectrum of post-infection and post-vaccination neurological, autoimmune, and inflammatory syndromes, including myocarditis, immune-mediated neuropathies, autoimmune cytopenias, systemic inflammatory responses [5–7], as well as temporal co-occurrence with cancer diagnoses, recurrences, or unexpectedly rapid disease trajectories [8–11]. These events have prompted extensive clinical investigation and underscore the capacity of vaccine-induced immune activation to perturb immune homeostasis in susceptible individuals. Importantly, many of these conditions are characterized by cytokine dysregulation, altered innate and adaptive immune signaling, and tissue-specific inflammatory responses; pathways that are also implicated in tumor initiation, progression, and immune surveillance. The present review focuses specifically on cancer-related observations within this broader context of post-vaccination immune perturbation.

After nearly six years since the pandemic was recognized in early 2020, the current world’s literature addressing COVID-19 infection or vaccination and cancer remains sparse, heterogeneous, and largely limited to case reports and small case series, insufficient to support definitive conclusions regarding causation or quantification of risk. Package inserts for COVID19 vaccines posted by the Food and Drug Administration (FDA) [12–15] specifically state that they have not been evaluated for carcinogenicity or genotoxicity, nor have they been studied after multiple vaccine doses and boosters or in combination with subsequent SARS-CoV-2 infection.

During the COVID pandemic, it was predicted that cancer rates would rise during and after COVID due to reduced screening and reduced access to treatment during the pandemic. However, rates of cancer among younger individuals for example with early onset colon cancer have been rising for two decades [16, 17]. Rates of cholangiocarcinoma and endometrial cancer have been rising as well. Cancer deaths exceeded 600,000 in US for 1st time in 2024 and in 2025 are predicted to rise as well [18]. As of the writing of this review, there are no published population studies in the US with mortality or cancer incidence follow-up beyond 42 days for outcomes after Covid infection versus no Covid infection or Covid vaccinated versus not Covid vaccinated. This is in part due to lack of good quality databases that would have such information. There is a National Cancer Institute (NCI)-funded Covid and Cancer Consortium (CCC) but it has not published on this topic specifically.

Against the backdrop of limited clinical evidence and incomplete preclinical toxicology, a recent study reported that SARS-CoV-2 mRNA vaccines may actually sensitize tumors to immune checkpoint blockade [19] prompting broad interpretation that COVID-19 mRNA vaccination may actually potentiate antitumor responses in patients with melanoma or non–small cell lung cancer (NSCLC) undergoing immune checkpoint inhibition. Moreover, in the analysis, mRNA vaccination was associated with increased Type I interferon signaling and elevated tumor PD-L1 expression. However, PD-L1 upregulation in the absence of checkpoint inhibitor therapy is generally associated with enhanced tumor immune evasion and resistance to T-cell–mediated cytotoxicity, raising questions about the biological interpretation of these findings. Although interferon-based therapies have established clinical utility in melanoma, the study did not provide comparative analyses between interferon treatment and the combination of mRNA vaccination with checkpoint blockade. Furthermore, the study did not address key limitations, alternative mechanistic explanations, or the broader clinical context necessary to fully interpret the reported effects.

This absence of evaluation of COVID19 vaccines for carcinogenicity or genotoxicity motivated a systematic review and synthesis of the available evidence from 2020–2025 concerning COVID-19 vaccination, SARS-CoV-2 infection, and cancer. Specifically, we sought to (i) categorize malignancies reported in temporal proximity to vaccination or infection, (ii) evaluate temporal and clinical patterns across tumor types for relevant signals among patients exposed to the COVID vaccines, and (iii) outline plausible immunologic and molecular mechanisms that could underlie these phenomena.

Across the published literature, we identified reports involving hematologic malignancies, including lymphomas and leukemias, solid tumors such as breast, lung, pancreatic, and glial cancers, virus-associated malignancies including Kaposi sarcoma and Merkel cell carcinoma, and rare entities such as sarcomas, melanomas, and adenoid cystic carcinomas. While the number of studies or their temporal association does not establish causation, understanding whether these associations represent coincidence, immune dysregulation, or a broader biologic effect linking infection, vaccination, and cancer development is now of pressing importance.

Importantly, regarding reported adverse events and potential risks, awareness of what has occurred, even if ultimately this proves to be extremely rare, is a necessary component of informed consent at a time when there is no longer a public health emergency from COVID-19. Cancer risk is likely based on heterogeneity among individuals, the impact of genetics, environment, and interacting social determinants of health that varies among individuals and this is an area where this article could form a foundation for future studies to refine individualized risk. As such, the goal of this article is to systematically synthesize and contextualize findings from the published literature regarding malignancies temporally associated with COVID-19 vaccination or SARS-CoV-2 infection, without attempting to estimate risk, establish causality, or inform individual clinical or vaccination decisions.

## RESULTS

This scoping review, covering the period of January 2020 until April 2025, was not designed to estimate cancer risk or incidence, nor to draw causal inferences, but rather to systematically assemble, categorize, and contextualize published reports of malignancies temporally associated with COVID-19 vaccination or SARS-CoV-2 infection. It identified 69 publications [8, 20–87] describing malignancies or malignant progression in temporal association with COVID-19 vaccination or SARS-CoV-2 infection, encompassing a total of 333 patients (excluding population-level studies [8, 20]. In addition, one population-level publication which offered a longitudinal assessment of cancer incidence across the pandemic and immediate post-pandemic period was identified [85]. Among the 69 studies, most reports were single-patient case reports or small series (55/69, 81%), with a small number of systematic or narrative reviews (3/69, 4.5%), mechanistic/experimental studies (2/69, 3%), and larger case series, multicenter, or database-level analyses (8/69, 12%) (Table 1). Consistent with an early signal-detection phase, the underlying evidence base is therefore heavily weighted toward documenting occurrences of potentially adverse events and hypothesis-generating case-level observations rather than population-based epidemiologic studies.

**Table 1:** Summary of reports linking COVID-19 vaccination or infection to cancer

Study type	*N*	% of Total (*N* = 69)	Comments
*Case reports*	50	72%	Dominant study type; mostly single-patient descriptions
*Case series*	5	7%	Typically 2-several patients
*Systematic/narrative reviews*	3	4%	Summaries or literature syntheses
*Cohort/retrospective/observational population studies*	8	12%	Larger-scale data (e.g., population cohort, single center cohort)
*Mechanistic/translational studies (tissue, organoids, mouse)*	3	4%	Experimental or preclinical mechanistic work

### Geographic distribution

Reports originated from a wide range of countries spanning Asia, Europe, the Middle East, Africa, and North and South America. The countries with the highest number of publications were Japan (*n* = 11) and the United States (*n* = 11), followed by China (*n* = 7) and Italy (*n* = 4). Additional single-patient cases or small series were identified from Spain, South Korea, Saudi Arabia, India, Nigeria, Brazil, Turkey, Singapore, Lebanon, Egypt, Bulgaria, Taiwan, Ukraine, Iran, Russia, Greece, Austria, Germany, Poland/Ukraine, as well as multi-institutional or international collaborations. This broad geographic distribution indicates that the reported temporal associations between COVID-19 vaccination or infection and oncologic events are not confined to a particular region or healthcare system but have been observed across diverse clinical settings and diagnostic infrastructures around the globe.

### Exposure types: Vaccination versus infection

Most publications identified in the search focused on oncologic events occurring after COVID-19 vaccination (56/69; 89%), with the remainder describing associations following SARS-CoV-2 infection (5/69; 7%), and SARS-CoV-2 infection with prior vaccination (7/69; 10%). One publication (1/69; 1%) did not explicitly specify whether the reported oncologic event followed vaccination, SARS-CoV-2 infection, or a combination of both exposures. These included case reports and mechanistic studies evaluating post-infectious tumor behavior, immune perturbation, or disease acceleration along with SARS-CoV-2 infection but in the absence of vaccination or associated with a SARS-CoV2 infection but with prior vaccination or boosting. The predominance of vaccination-associated case reports may reflect reporting patterns rather than comparative biological risk, and the available data lack sufficient individual-level detail to determine whether or how oncologic responses differ between infection or vaccination.

**Figure 1:**
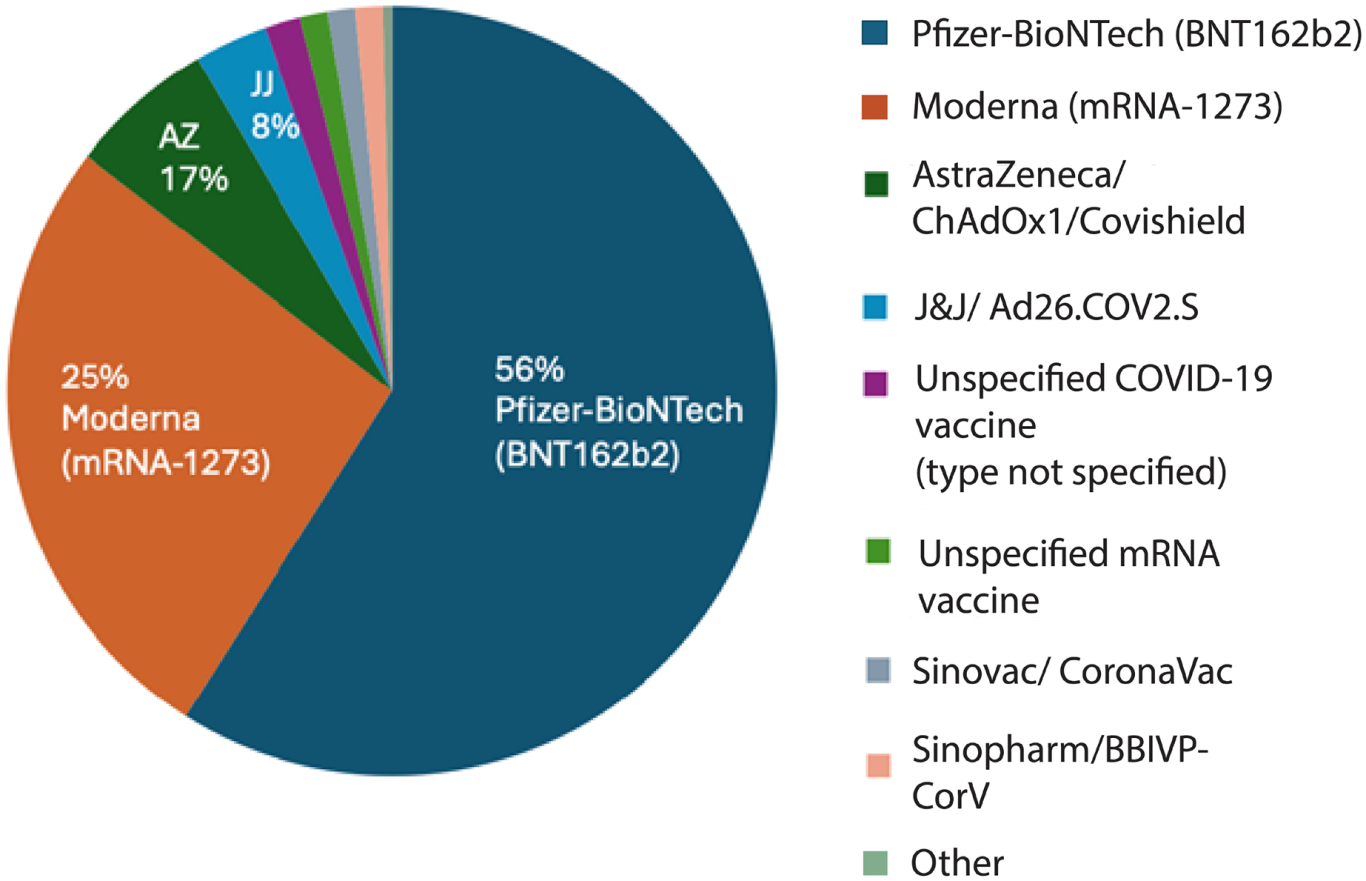
Distribution of reported malignancies by COVID-19 vaccine type. Distribution of vaccine formulations among vaccinated patients with reported cancer following COVID-19 immunization. Most cases involved Pfizer-BioNTech (BNT162b2; 56%) and Moderna (mRNA-1273; 25%) vaccines, followed by AstraZeneca/ChAdOx1 (Covishield; 17%) and Johnson & Johnson/Ad26.COV2.S (8%). A small fraction of reports involved Sinovac (CoronaVac), Sinopharm (BBIBP-CorV), or other inactivated vaccines, as well as unspecified mRNA or COVID-19 vaccine types. The predominance of mRNA vaccines reflects their widespread global use during the study period.

Across the published literature, reported vaccine formulations and exposure types were heterogeneous but could be grouped into broad platform categories (Figure 1). Among vaccine-related reports, the majority involved mRNA vaccines, with approximately 56% following the Pfizer-BioNTech vaccine (BNT162b2) and 25% following the Moderna vaccine (mRNA-1273). An additional 5% involved patients who had received both Pfizer and Moderna products across different doses. Adenovirus vector vaccines represented the next largest category, including AstraZeneca (ChAdOx1/Covishield) (5.8%), Johnson & Johnson (Ad26.COV2.S) (2.9%) and the Russian, Sputnik-V (1.4%). Inactivated vaccines (e.g., Sinopharm BBIBP-CorV, CoronaVac, or other formulations) and studies in which the specific vaccine type was not reported were least represented (2.6% and 1.1%, respectively). This distribution indicates that the published literature is heavily weighted toward mRNA vaccine platforms, particularly Pfizer-BioNTech and Moderna, which together account for the vast majority of vaccine-associated reports. This pattern closely mirrors global vaccination practices where mRNA vaccines were most widely deployed. The relatively smaller representation of adenoviral vector vaccines and inactivated platforms likely reflects both their more limited use in certain regions and differential reporting practices, rather than a comparative assessment of biological risk.

### Cancer types and clinical spectrum

**Figure 2:**
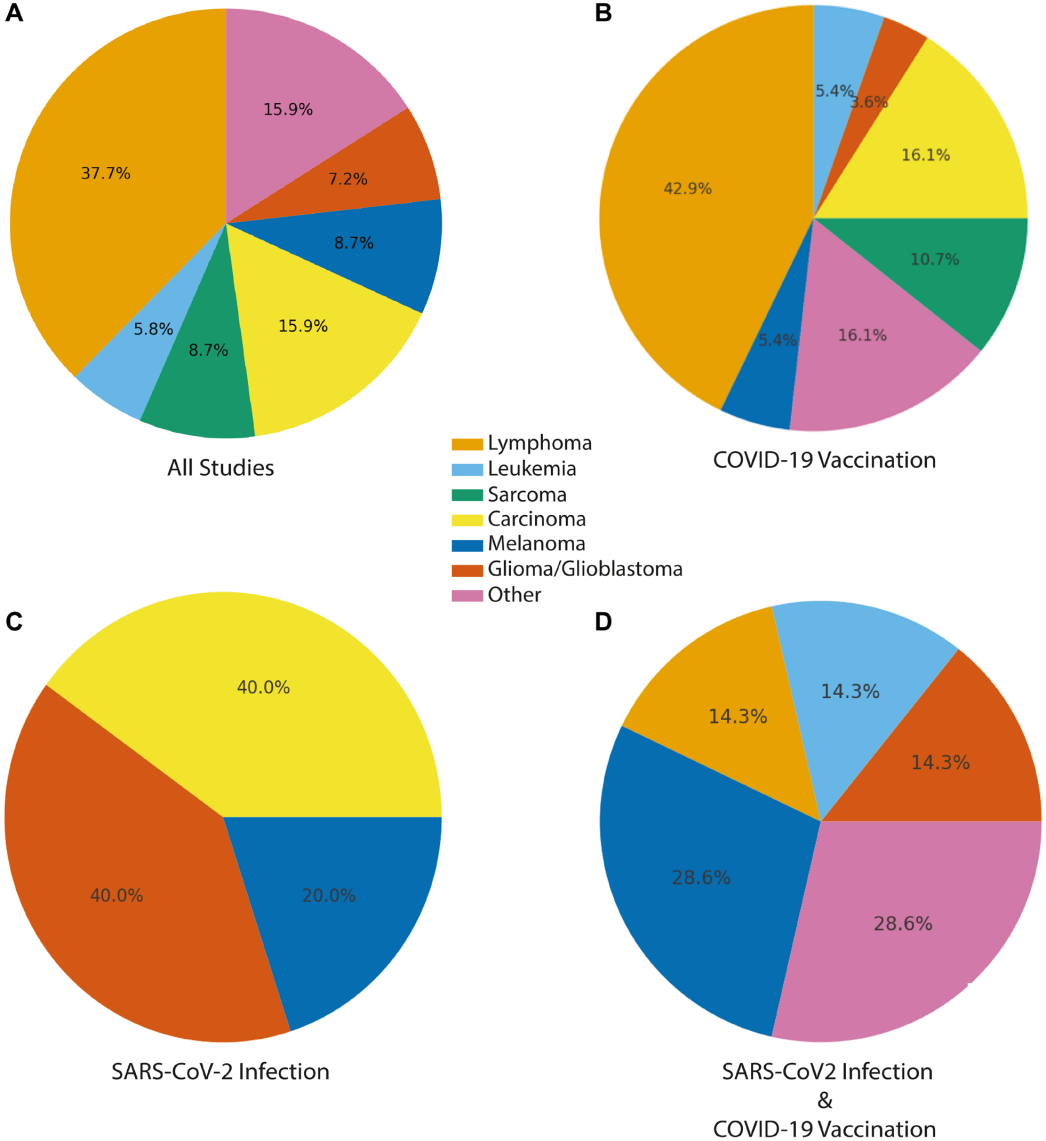
Distribution of post-vaccination and post-infection malignancies by tumor type. Distribution of reports with malignancy or tumor-like lesions temporally associated with COVID-19 vaccination, SARS-CoV-2 infection, or SARS-CoV-2 infection and vaccination. Pie charts depict the proportional representation of major cancer categories observed. (**A**) Accross all studies. (**B**) COVID-19 vaccination, (**C**) SARS-CoV-2 infection, and (**D**) combined SARS-CoV-2 infection and COVID-19 vaccination. Cancer types were consolidated into seven high-level categories. Carcinoma includes: breast cancer, prostate cancer, colon cancer, pancreatic cancer, lung cancer, Merkel cell carcinoma, GI neoplasia/polyposis. Lymphoma also includes lymphoid neoplasms, cutaneous lymphoproliferative disorders, lymphoproliferative disorder. Other includes benign tumors, pseudotumors, mixed tumors, heart tumors, inflammatory and non-specific tumors (e.g., myofibroblastic).

Approximately 43% (30/69) of publications reported lymphoid malignancies, encompassing both lymphomas and leukemias (Figure 2 and Table 2). These included a wide spectrum of lymphoid neoplasms such as diffuse large B-cell lymphoma (DLBCL), various T-cell lymphomas (e.g., angioimmunoblastic T-cell lymphoma, subcutaneous panniculitis-like T-cell lymphoma), chronic lymphocytic leukemia/small lymphocytic lymphoma (CLL/SLL), and cutaneous T-cell lymphomas (CTCL). Several reports emphasized unexpectedly rapid progression, atypical presentations, or unusually aggressive courses of disease.

**Table 2:** Clinicopathologic spectrum of lymphomas in post-vaccination reports

Lineage	Subtypes	Key features
*T-cell lymphomas*	CTCL, LyP, ALCL, AITL, SPTCL, TFH-type, PCGDTCL, T-ALL, T- cellNOS	Dominated by cutaneous and TFH-derived entities; several at injection sites; many indolentor self-resolving (CD30^+^).
*B-cell lymphomas*	DLBCL, Follicular, MZL, CLL	Primarily DLBCL; often nodal or axillary post-mRNAvaccine; typically de novo; most treated with R-CHOP.
*NK/NK-T-cell lymphomas*	ENKL (nasal-type), NK/T overlap	EBV^+^ nasal lesions; one partial response to SMILE + radiation; suggest EBV reactivation.
*Mixed/Unspecified LPDs*	Large “unspecified/other” cohort from systematic review (Cui 2024) and PCLDs	Aggregate data without cell-lineage resolution; largely literature or registry series.

**Figure 3:**
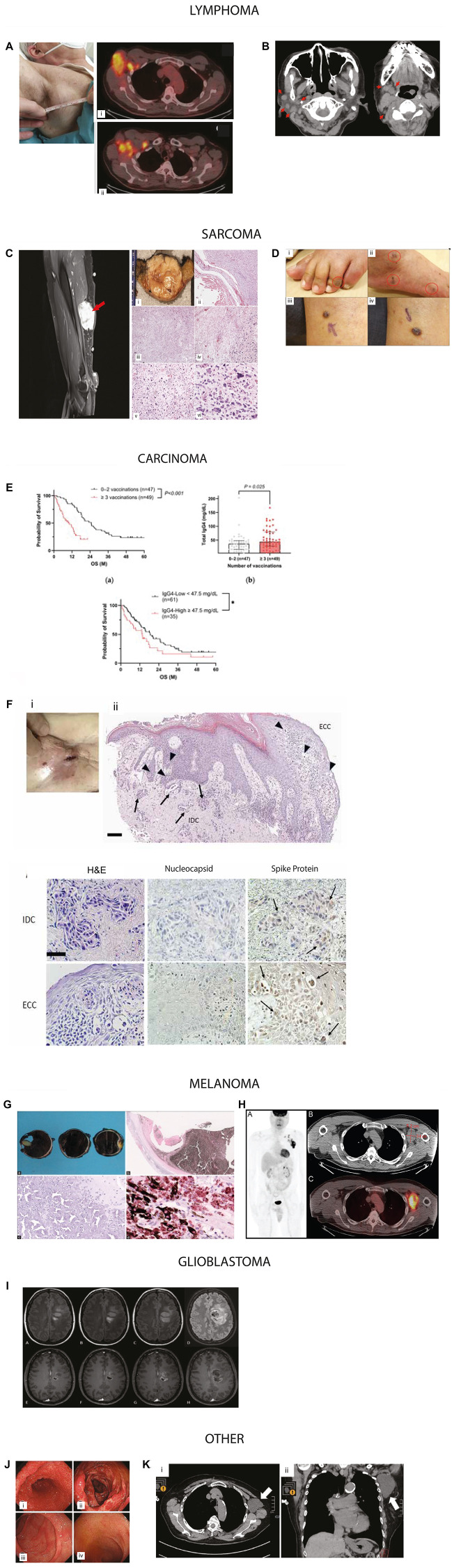
Representative examples of cancers reported in temporal association with COVID-19 vaccination. Figures were reproduced with permissions (Supplementary Table 1). Lymphoma: (**A**) Axillary adenopathy and i) 18-FDG-PET/CT at baseline in the right axillary adenopathy mass and ii) in multiple axillary adenopathies and subsequent NHL diagnosis following vaccination. Image reproduced from Cavanna et al., Medicina, 2023. © MDPI. (**B**) Temporal mass after her first BNT162b2 dose, with persistent lymphadenopathy on imaging. Axial computed tomography image shows (i, ii) submandibular and jugular regions. Image reproduced from Sekizawa et al., Front Med, 2022. © Frontiers. Sarcoma (**C**) High-grade sarcoma arising near injection site. A 6-cm right upper-arm mass after second Moderna dose, near the prior injection site; pathology confirmed high-grade sarcoma. Image adapted with permission from Bae et al., Cureus, 2023 © Springer Nature. (**D**) Classic cutaneous Kaposi’s sarcoma adapted from Li et al. Front Med, 2022 © Frontiers. A 79-year-old man developed violaceous papules on the legs after the first ChAdOx1 vaccine dose; biopsy confirmed KS. Treatment included radiotherapy and doxorubicin. Clinical images of Kaposi sarcoma (i) with dark brown macules over the left foot, (ii) the right foot and larger reddish erythematous papules on his left calf (iii, iv). Carcinoma (**E**) In a 96-patient cohort, repeated booster vaccination correlated with poorer overall survival and elevated IgG4 level of pancreatic ductal adenocarcinoma. Kaplan–Meier analysis of 96 PC patients with known vaccination history and measured IgG4 levels, total IgG4 levels by number of vaccinations, and Kaplan–Meier analysis in PC patients by IgG4 levels. Image adapted with permission from Abue et al. Cancers 2025 © MDPI. (**F**) A case of metastatic breast carcinoma to the skin expressing SARS-CoV-2 spike protein. Histopathology of skin metasstatis along with IHC for nucleocapsid and Spike protein. Images adapted from Sano, S., J. Derm Sci, 2025. © Elsevier. Melanoma (**G**) Gross examination of specimen shows extensive intraocular hemorrhage involving both anterior and posterior chambers, accompanied by complete retinal detachment. H&E stained section shows severely degenerated, necrotic melanocytic lesion located with widespread necrosis within the melanocytic tumor. SOX10 IHC confirms melanocytic cells containing cytoplasmic melanin, interspersed among numerous SOX10-negative melanophages. Image adapted with permission from Wagle et al. Indian J Ophthalmo 2022 © Wolters Kluwer. (**H**) Maximum-intensity projection PET image shows markedly increased radiotracer uptake within the left axillary and supraclavicular lymph nodes. Representative axial CT and corresponding fused PET/CT images highlight the dominant nodal conglomerate. The patient had received a COVID-19 vaccination in the left upper arm within two months prior to imaging. Image adapted from Gullotti et al. Radiol Case Rep. 2022 © Elsevier. Glioblastoma (**I**) Two patients (ages 40 and 31) presented with new neurologic deficits and frontal-lobe masses shortly after mRNA vaccination. Image adapted from O’Sullivan et al. J of Neurology. 2021 © Elsevier. Other (**J**) Gastrointestinal polyposis identified following COVID-19 vaccination. Image adapted with permission from Kim et al. Clin Endosc 2024 © Korean Society of Gastrointestinal Endoscopy (**K**) Axillary lymphangioma in an 80-year-old woman three months after her second Pfizer-BioNTech dose; imaging showed a cystic lymphangioma. Image adapted with permission from Sasa et al. Surg Case Rep 2022 © Springer Nature.

Solid tumors accounted for 41% of publications (28/69) and represented a diverse group of malignancies, including melanoma, breast cancer, lung cancer, glioblastoma and other glial tumors, sarcomas, and various organ-specific carcinomas, such as pancreatic cancer (Figures 2 and 3). In multiple reports, the authors described unusually rapid onset, short-latency recurrence, or aggressive clinical progression for tumor types such as pancreatic adenocarcinoma and glioblastoma; features that are atypical for these cancers highlighted as notable temporal observations.

A subset of reports described tumor formation or recurrence at or near vaccine injection sites, the deltoid region, axilla, or draining lymphatic basins, including cases where axillary lymphadenopathy coincided with solid-tumor metastasis. Virus-associated malignancies such as Kaposi sarcoma, Merkel cell carcinoma, and EBV-positive lymphomas were also identified across several reports. The remaining 16% of publications (11/69) were categorized as other or unspecified, which included mixed or indeterminate cases, non-malignant proliferations, studies referencing “cancer”, “tumor”, or “malignancy” without definitive histopathologic classification, and population-level analyses in which tumor type was not explicitly delineated.

### Specific examples of cancers and their association with COVID vaccination

#### Lymphoma

Cavanna et al. [26] reports the review of a series of eight patients who developed Non-Hodgkin’s Lymphoma after COVID-19 vaccination (Table 3), including four males and four women. Five patients were vaccinated with the BNT162b2 vaccine (Pfizer), one with the ChAdOx1 nCOV-19 vaccine (AstraZeneca, Cambridge, UK), one with mRNA 1273/Spikevax (ModernaTX) and one patient with the recombinant replication-incompetent adenovirus type 26 (Ad26) viral-vector-based COVID-19 vaccine (Janssen Pharmaceuticals, Beerse, Belgium). One of the NHL cases presented with large right axillary adenopathy shortly after COVID-19 vaccination (Figure 3A).

**Table 3:** Summary of case series describing malignant lymphoma following mRNA COVID-19 vaccination

Case N	Gender/Age (Year)	Time from Vaccination to Onset of Lymphoproliferative Disorder	Histopathological Examination	Type of COVID-19 Vaccine	Site and Diameter of Lymphadenopathy	Treatment of Lymphoma
1	M/67	1 day after 1 dose	DLBCL	BNT162b2	Left axilla 6.0 cm	Chemotherapy plus rituximab
2	F/80	2 days after 1 dose	DLBCL	BNT162b2	Left axilla 4.1 cm	Chemotherapy plus rituximab
3	F/58	7 days after 2 dose	DLBCL	BNT162b2	Left cervical area 4 cm	Radical surgery plus radiotherapy
4	M/53	3 days after 1 dose	Extranodal NK/T-cell lymphoma	BNT162b2	Erosive lesions upper lip up to 5 mm	Chemotherapy plus radiotherapy
5	M/51	7 days after 1 dose	EBV-positive DLBCL	ChAdox1 nCOV-19	Mediastinal mass 5 cm	Rituximab
6	F/28	“A few days after 1 dose”	SPTCL	Ad26 viral-vector- based	Injection site, upper arm	Cyclosporine plus prednisone
7	F/80	1 day after 1 dose	EMZL	BNT162b2	Right temporal mass	No treatment
8	M/76	10 days after the booster dose	PC-ALCL	mRNA-1273^*^	Right arm upper-external surface 6 cm	No treatment

Table reproduced from Cavanna et al., Medicina, 2023. © MDPI. Abbreviations: ALCL: anaplastic large-cell lymphoma; DLBCL: diffuse large B-cell lymphoma; EBV: Epstein-Barr virus; EMZL: extranodal marginal zone lymphoma; PC-ALCL: primary cutaneous anaplastic large-cell lymphoma; SPTCL: subcutaneous panniculitis-like T-cell lymphoma. ^*^The two previous vaccination doses were BNT162b2.)

Sekizawa et al. [28] describe a case of marginal zone B-Cell lymphoma in an 80-year-old Japanese woman who presented with a right temporal mass that appeared the morning after she was administered her first mRNA COVID-19 vaccination (BNT162b2) (Figure 3B). The mass gradually decreased in size but persisted over 6 weeks after her first vaccination (3 weeks after her second vaccination). At her first visit, ultrasound revealed the size of the mass to be 28.5 Å~ 5.7 mm, and computed tomography revealed multiple lymphadenopathies in the right parotid, submandibular, jugular, and supraclavicular regions. This case brings up the possibility that an initial mass may not be composed entirely of cancer cells and may have an element of a host response that may limit the progression depending on immune or other factors. In this case, the patient had marginal zone B-cell lymphoma after BNT162B2 COVID-19 vaccination.

#### Sarcoma

Bae et al. [21] reported the development of high grade sarcoma after the second dose of the Moderna vaccine. A 73-year-old female with a past medical history of hypertension, hyperlipidemia, and renal angiomyolipoma status post resection in 2019 presented with worsening right upper arm swelling for the past two weeks. She noticed the swelling two to four days after receiving her second dose of the Moderna vaccine within 1 cm from the prior injection site. Physical examination was remarkable for a 6 cm, circular, mobile, soft mass present in the right upper arm. (Figure 3C). Li et al. [23] reported the development of classic cutaneous Kaposi’s sarcoma in a 79-year-old male following the first dose of the ChAdOx1 nCov-19 vaccine, without prior SARS-CoV-2 infection or history of HIV infection. The patient developed multiple reddish-blue papules on his legs and feet, confirmed as KS through histopathology (Figure 3D). Treatment included radiotherapy and sequential chemotherapy with doxorubicin. The potential reactivation of latent HHV-8 by the vaccine is suggested through mechanisms involving the SARS-CoV-2 spike protein and adenovirus vector, which may induce immune responses and inflammatory pathways.

#### Carcinoma

Abue et al. [32] describe a case series of 96 patients with the diagnosis of pancreatic ductal adenocarcinoma (Figure 3E). Repeated COVID-19 booster vaccinations were associated with worse overall survival in the patients with pancreatic cancer. Analysis revealed that high levels of IgG4, induced by vaccination, correlate with a poor prognosis. Sano [36] described an 85-year-old woman who presented with an asymptomatic skin lesion in the right chest within one month immediately after the 6th dose of (Pfizer-BioNTech) vaccination. The patient had been diagnosed with right breast cancer two years prior and underwent partial mastectomy, hormone therapies, and was deemed to be in remission. The lesion was confirmed as a skin metastasis deemed to have developed through potential local recurrence at surgical margins (Figure 3F).

#### Melanoma

Wagle et al. [56] described a 49-year-old Indian male who developed rapidly progressive vision loss one day after receiving a second dose of the BNT162b2 mRNA COVID-19 vaccine (Pfizer–BioNTech, USA). Ophthalmologic exam revealed secondary angle-closure glaucoma, bullous retinal detachment, and extensive intraocular hemorrhage. Ocular imaging and confirmed magnetic resonance imaging (MRI) revealed an ill-defined heterogeneous subretinal lesion, with histopathology confirming necrotic uveal melanoma (Figure 3G). Gullotti et al. [55] also described an otherwise healthy 53-year-old man who presented with ipsilateral axillary lymphadenopathy and associated discomfort shortly after receiving a COVID-19 vaccine. Fine-needle aspiration performed within two months of vaccination revealed metastatic melanoma, and subsequent 18F-FDG PET/CT imaging demonstrated intensely hypermetabolic axillary and supraclavicular lymphadenopathy without identification of a primary tumor (Figure 3H).

#### Glioblastoma

Tosun et al. [29] reported a 40-year-old man presenting with left hemiparesis. He had received COVID-19 vaccination 3 weeks before. Brain MRI showed a central cystic necrotic lesion with indistinct borders in the right frontal lobe as mild peripheral contrast enhancement surrounded by smaller nodular lesions. O Sullivan et al. [84] also describe a 31-year-old female who first noted a slight weakness of her right leg about 7 days after receiving the first dose of a COVID-19 mRNA vaccine (Comirnaty^®^BioNTech Manufacturing GmbH, Germany). She initially reported slight drowsiness and headache without fever following vaccination, which resolved within 24 h. Following the administration of the second intramuscular dose of the vaccination, 21 days after the first, the preexisting weakness of the right leg rapidly worsened and was accompanied by severe headache and night chills. Neurological examination on day 28 showed a mild central paresis of the right leg and numbness of the plantar surface of the foot (Figure 3I).

#### Other

Kim et al. [31] describe two cases of gastrointestinal polyposis (Cronkhite–Canada syndrome) shortly after administration of an mRNA booster vaccine for COVID19. Both showed numerous erythematous gastric and colonic polyps with villous atrophy throughout the small intestine (Figure 3J). The authors note that the timing, autoimmune features, and steroid responsiveness raise the possibility that mRNA vaccination may trigger Cronkhite–Canada syndrome in genetically susceptible individuals, warranting clinical vigilance. Sasa et al. [33] report on axillary lymphangioma following COVID-19 in a Japanese woman in her 80s who received a second injection of the Pfizer-BioNTech COVID-19 vaccine in her left deltoid muscle in 2021 (Figure 3K). She had a history of right breast cancer (T1N0M0) and had undergone breast-conserving surgery and sentinel node biopsy in her 70’s. Postoperative follow-up examinations were continued, and no sign of recurrence, including in the left axial region, was observed until 2021. There was no evidence of trauma to the left axial region. Her early adverse reaction following vaccination was mild pain at the inoculation site on the day of vaccination and the following day. However, 3 months after the second vaccination, she noticed left axillary swelling.

### *De novo* disease versus recurrence or progression

Most publications, including all the examples above, described de novo malignancies or apparent “unmasking” or activation of previously subclinical disease. A smaller subset focused predominantly on recurrence, progression, or metastatic reactivation in patients with a documented cancer history. An additional 13 publications reported mixed cohorts, including both new diagnoses and recurrences or provided explicit quantification of both categories. Only one publication did not clearly distinguish between new-onset and recurrent disease.

Taken together, these patterns indicate that the observed signal in the literature is not restricted to recurrence or flare of known malignancies. Rather, a substantial proportion of reports involve first-time cancer diagnoses temporally associated with COVID-19 vaccination or SARS-CoV-2 infection, highlighting the need to evaluate potential mechanisms that could contribute to disease initiation, unmasking, or acceleration.

### Timing of onset

Across the included studies, the timing of cancer onset following COVID-19 vaccination varied substantially, indicating that latency was not confined to a single early window. Approximately half of the case reports described diagnoses occurring within 2–4 weeks of vaccination, with some reported as early as 7–14 days. However, many reports also documented longer intervals, including diagnoses at 2–3 months, 4–6 months, and beyond eight months after vaccination. Importantly, reports with short intervals are inherently more likely to be recognized and published as temporally notable.

In addition, in many reports describing diagnoses within the first month, the event occurred after a second dose or booster, complicating attribution to any specific exposure and precluding definition of a uniform latency period. Multicenter analyses frequently characterized latency as variable, spanning weeks to months, and several reviews or population-level studies reported mean onset intervals of approximately 8–9 weeks.

Tumor growth rates vary significantly among tumor types from the fastest growing lymphomas and leukemias to slower growing solid tumors [87–92]. Accordingly, while a subset of published cases report diagnoses within weeks of vaccination, the broader literature reflects a continuum of reported latencies over several months, often in the context of cumulative exposure. These observations are therefore best interpreted as descriptive and hypothesis-generating, underscoring the need for standardized latency definitions and systematic evaluation in appropriately controlled studies.

### Population-level and registry-based studies

Three large-scale population-level analyses provided broader epidemiologic context to complement the case-based literature. Two retrospective population-level investigations, one in Italy [20] and one in South Korea [8], quantified cancer incidence and mortality trends among vaccinated populations. Kim et al. [8] analyzed approximately 8.4 million individuals between 2021 and 2023 to assess 1-year cumulative cancer incidence following COVID-19 vaccination using the South Korean National Health Insurance Service database. The authors reported statistically significant associations between vaccination and six specific cancers, including thyroid (HR 1.35), gastric (HR 1.34), colorectal (HR 1.28), lung (HR 1.53), breast (HR 1.20), and prostate cancer (HR 1.69) using propensity score matching and multivariable Cox proportional hazards models. Associations varied by vaccine platform, with mRNA vaccines linked to thyroid, colorectal, lung, and breast cancers, and cDNA/adenoviral vaccines associated with thyroid, gastric, colorectal, lung, and prostate cancers; heterologous vaccination was associated with thyroid and breast cancer. Stratified analyses suggested effect modification by sex and age, and booster-dose analyses identified increased risks for gastric and pancreatic cancer. The authors emphasized that despite adjustment for measured confounders, residual confounding, detection bias, and limited follow-up preclude causal inference, and that the findings should be interpreted as epidemiologic associations warranting further study rather than evidence of vaccine-induced cancer risk.

Acuti Martellucci et al. [20] evaluated associations between SARS-CoV-2 vaccination, all-cause mortality, and hospitalization for cancer using multivariable Cox proportional hazards models in a population-wide retrospective cohort study of 296,015 residents of the Pescara province in Italy followed for up to 30 months (June 2021–December 2023). Hospitalization for cancer of any site was found to be modestly higher among vaccinated individuals compared to unvaccinated (≥1 dose: HR 1.23, 95% CI 1.11–1.37; ≥3 doses: HR 1.09, 95% CI 1.02–1.16), with site-specific increases observed primarily for colorectal (HR 1.35), breast (HR 1.54), and bladder cancer (HR 1.62) after ≥1 dose, and for breast and bladder cancer after ≥3 doses. These associations varied by sex, prior SARS-CoV-2 infection, vaccine type, and lag time between vaccination and outcome, and were attenuated or reversed when a longer minimum latency of 365 days was applied. The analyses adjusted for age, sex, prior SARS-CoV-2 infection, and multiple comorbidities (including cardiovascular disease, diabetes, COPD, kidney disease, and prior cancer), but lacked information on key behavioral and healthcare-utilization confounders such as smoking, cancer screening, and healthcare-seeking behavior. The authors explicitly note that residual confounding, healthy-vaccine bias, detection bias, and reliance on hospitalization data as a proxy for cancer incidence limit causal interpretation, and they characterize the findings as preliminary and hypothesis-generating rather than evidence of vaccine-induced cancer risk. Both studies provide early, population-level associations rather than causal estimates and underscore the importance of long-term follow-up and molecular correlation to distinguish true biological effects from health-system or behavioral confounders.

**Figure 4:**
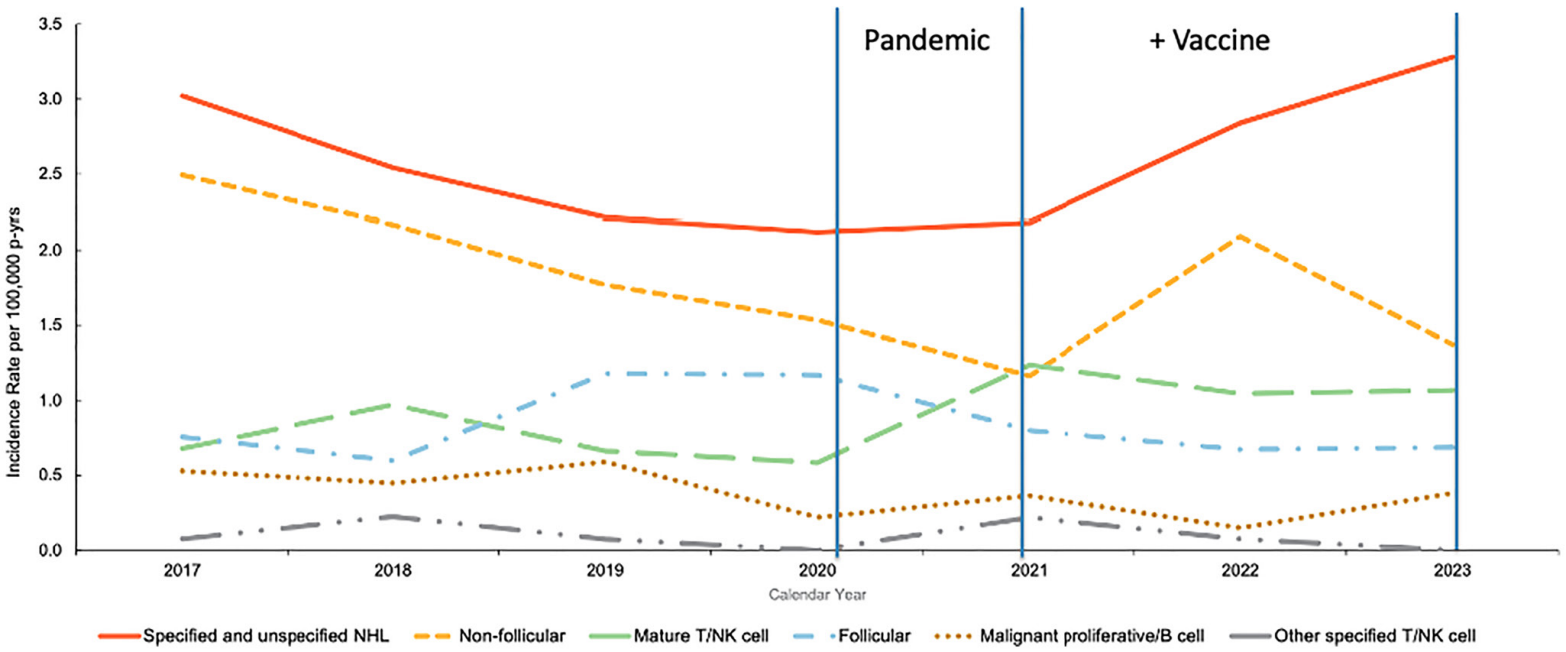
Annual incidence rates of non-Hodgkin lymphoma (NHL) subtypes among active-component U.S. service members, 2017–2023. Figure adapted from Russell et al. [85] using Defense Medical Surveillance System data demonstrating rise in specified/unspecified NHL and mature T/NK-cell subtypes. Vertical lines denote key timepoints: the onset of the COVID-19 pandemic (early 2020) and the beginning of the Department of Defense vaccine mandate (late 2020–early 2021).

In addition to these population-level studies, a recent US Armed Forces Health Surveillance Division (AFHSD) report was also identified that presented population-level analyses of non-Hodgkin lymphoma (NHL) incidence among active-duty U.S. service members from 2017 through 2023 [85]. The U.S. Department of Defense (DoD) mandated COVID-19 vaccination for all active-duty service members (~1.3 million) beginning in late 2020, with near-universal compliance achieved by mid-2020; this cohort offers a rare longitudinal view of cancer incidence across this transition. Using data from the Defense Medical Surveillance System (DMSS), the authors calculated annual incidence rates (IRs) per 100,000 person-years and categorized cases by lymphoma subtype and the 2017–2020 interval largely represents a pre-vaccine baseline, whereas 2021–2023 reflects a fully vaccinated, post-pandemic cancer incidence [86] (Figure 4).

Notably, a rise in mature T/NK-cell lymphomas began across the 2020–2021 transition which spans the period of COVID-19 infection and the beginning of widespread vaccination in the military. Beginning in 2021, a ~50% increase in specified/unspecified and non-follicular NHL subtypes, accompanied by a persistently elevated incidence of mature T/NK-cell lymphomas relative to pre-pandemic years was observed. Notably, the authors did not attribute the observed changes in NHL incidence to vaccination or infection, and the analysis was not designed to establish causality at the individual level. Changes in diagnostic practices, healthcare access and utilization, and pandemic-related disruptions to routine medical care cannot be excluded from this time-trend analysis as with others conducted during the pandemic period. However, these findings provide descriptive temporal trends within a unique and highly structured population, providing an epidemiologic framework for future controlled analyses.

Taken together, these population-level analyses combined with the case-based literature indicate that a cancer signal warrants further prospective evaluation to determine whether COVID-19 vaccination confers any measurable cancer risk or merely reflects surveillance and reporting biases.

## MATERIALS AND METHODS

A comprehensive search of the world’s literature was conducted using PubMed, Scopus, Web of Science, Google Scholar, and React19 between January 2020 and April 2025. Eligible publications included case reports, case series, cohort or population-level analyses, systematic reviews, and mechanistic or preclinical studies that described either (i) new-onset, recurrent, or rapidly progressive malignancy temporally associated with COVID-19 vaccination or SARS-CoV-2 infection, or (ii) experimental evidence implicating vaccine or infection-induced immune perturbations in oncogenic, proliferative, or metastatic processes.

Initial searches in PubMed using conventional keyword combinations such as “COVID-19 vaccine and cancer,” “vaccination and cancer,” “COVID-19 vaccine and tumor,” or cancer-specific terms paired with “COVID-19 vaccine” yielded little to no indexed results. Even when known case reports were used as anchors for “similar articles,” PubMed returned no related entries. This highlighted a structural limitation in standard indexing pathways and necessitated a broader, more strategic search approach.

A general web-based search (e.g., Google) returned an autogenerated AI summary when queried for the terms “COVID vaccine and cancer” indicating that major health agencies, including the Centers for Disease Control and Prevention (CDC) and the National Cancer Institute (NCI), recommend COVID-19 vaccination for individuals with cancer and assert that the vaccines are considered safe for this population and are not believed to cause cancer or precipitate recurrence. Therefore, an expanded search strategy was implemented using combinations of general and tumor-specific terms, including: “COVID-19,” “SARS-CoV-2,” “spike,” “vaccination,” “vaccine,” “tumor,” “cancer,” “neoplasia,” “malignancy,” “recurrence,” “progression,” “lymphoma,” “leukemia,” “melanoma,” “glioma,” “adenocarcinoma,” “sarcoma,” “Kaposi,” “Merkel cell,” “cardiac”, and related descriptors. Databases were searched using Boolean operators, varied term order, and MeSH/non-MeSH variants to overcome incomplete tagging or atypical indexing of case reports.

Studies were included irrespective of patient age, sex, geographic region, cancer histology, or vaccine platform (mRNA, viral-vector, or inactivated). Exclusion criteria consisted of commentaries, opinion correspondence, purely theoretical articles lacking clinical or experimental data, and duplicate case entries across publications. Studies labeled as “COVID-associated” or “COVID-related”, particularly for cardiac tumors ultimately described patients who tested negative for SARS-CoV-2 [93]. For methodological consistency, we excluded such reports from the infection-focused section of the analysis, as the absence of virologic confirmation precludes attributing the observed malignancy to active or recent infection. Reference lists of systematic reviews and larger case compilations were manually screened to identify secondary citations not captured in the primary search. All included articles were independently cross-referenced in PubMed when possible, to confirm indexing status and ensure completeness.

## Mechanistic hypotheses linking COVID-19 vaccination or infection to oncogenic events

The case studies and emerging population-level data described above may represent an early signal of a possible association between vaccination or infection and cancer that warrants further investigation. This raises the question: if there is an association, what might be the mechanistic basis for it?

Viruses can cause cancer [94–97]. The relationship between viral infection and cancer has been well-documented for Human Papilloma Virus (HPV) that causes cervical cancer, head and neck cancer, as well as anal cancer that is increased among HIV-infected individuals. Hepatitis B Virus (HBV) and Hepatitis C Virus (HCV) cause liver cancer. Epstein Barr Virus (EBV) causes nasopharyngeal cancer, Burkitt’s Lymphoma, and other cancers. The human herpes virus KSHV/HHV-8 causes Kaposi’s sarcoma, the Human T-cell Leukemia Virus (HTLV-1) causes adult T-cell leukemia or lymphoma, and the Merkle Cell Virus (MCV) causes Merkle cell skin cancer. Several viruses are suspected of causing cancer including SV40 (mesothelioma, primary brain and bone cancers, among others) and HCMV (glioblastoma, medulloblastoma, breast, colon and prostate cancer). HIV is strongly associated with Kaposi’s sarcoma, cervical cancer, lymphoma, anal cancer, and other malignancies, largely though immunosuppression and co-infection with oncogenic viruses. It has been known for decades that viral proteins target host tumor suppressors such as p53 and Rb, suppress the immune system, and activate oncogenic signals.

In addition, the COVID mRNA vaccines work by instructing the target cells to produce the SARS-CoV-2 spike protein. This occurs by introducing a synthetic, modified mRNA (mod-mRNA) which incorporates non-natural pseudouridine into its coding region to prolongs the stability of the mRNA beyond that of natural mRNA. Introduction of the mod-RNA is accomplished using lipid-based transfection in the form of lipid nanoparticles (LNPs). The result is highly efficient transfection of the mod-mRNA into target cells with biochemical and pharmacological behavior different from naturally occurring mRNA. Consequently, the mod-RNA is transcribed into the foreign spike protein (as well as other frameshifted protein products), which elicits a robust immune response [98–102]. Given the stability of pseudouridine modified mRNA, along with the residual DNA in the mRNA vaccine formulations [103–108], the mRNA vaccines are delivering exogenous genetic material (DNA and RNA (in the form of engineered nucleic acids)) into a patient’s cells. The COVID19 mRNA vaccines produce Spike protein that is encoded by a stable mRNA and has been found to be long-lived in the human body [109, 110]. These nucleic acid elements have been reported to contribute to Post-Covid Vaccine Syndrome (PCVS/PVS) [110, 111]. Thus, these vaccines fit the definition of gene therapy [112, 113]. Despite this, there are efforts by the EU to modify the definition of gene therapy to exempt mRNA vaccines from this category [114].

While there are no studies demonstrating a direct causal mechanism by which the mRNA vaccines induce cancer, cumulative molecular effects from persistent spike protein [115, 116], the immune activation and inflammation from repeated vaccination [117–119], or the potential for genomic integration events [120] might contribute to events that could in theory manifest in cancers following vaccination or infection. Given the rapid onset of aggressive and rare tumors from the literature, cancers arising weeks to months after vaccination would be perhaps more consistent with mechanisms involving tumor promotion rather tumor initiation *per se*. However, mechanisms involving initiation are also considered. Here we present least three biologically plausible mechanisms that might explain an association between COVID-19 vaccination and cancer; two of them overlapping with covid infection, immune dysfunction and spike protein biology, and reactions due to DNA impurities restricted to vaccination.

## Immune dysregulation

**Figure 5:**
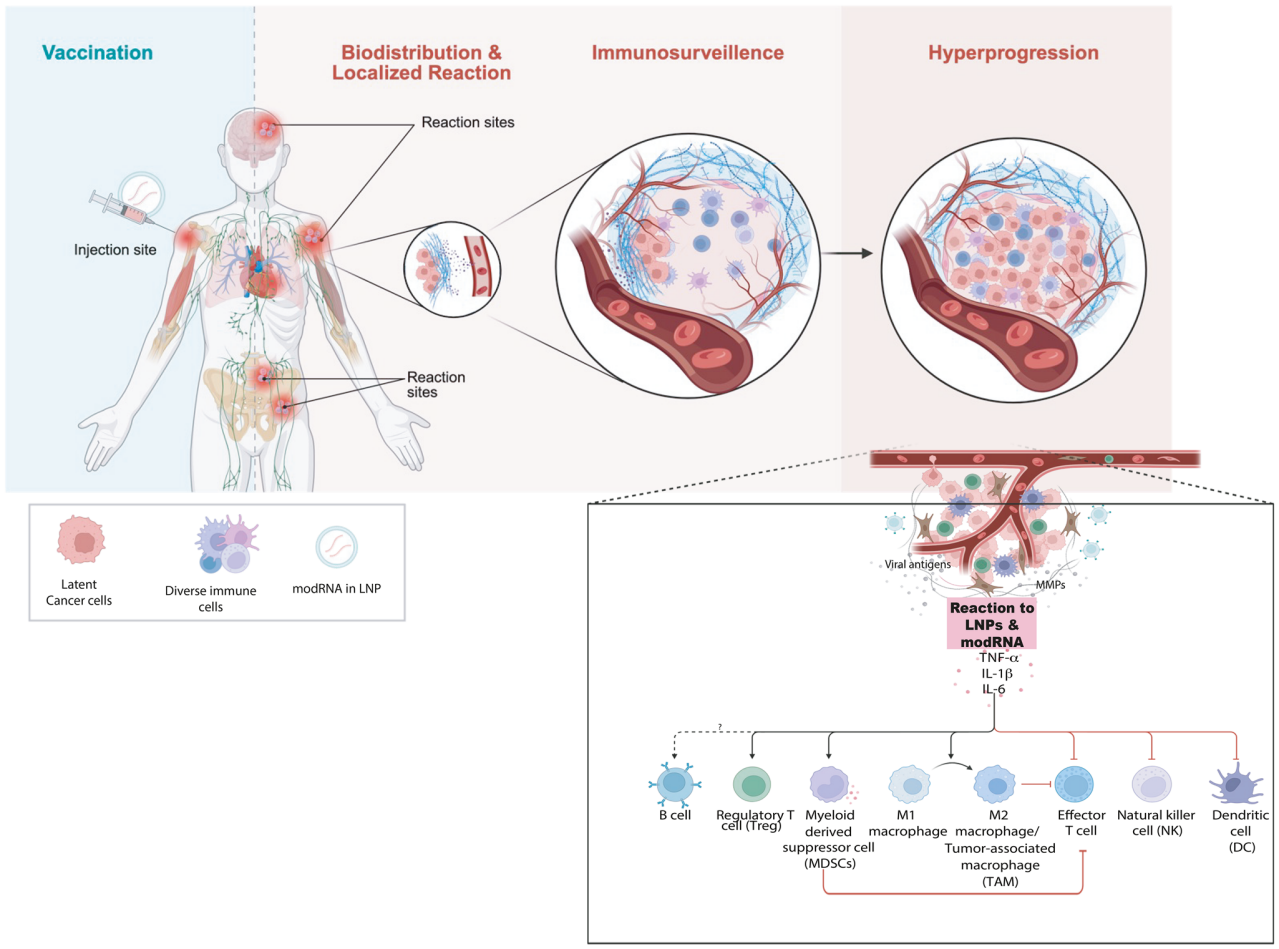
Proposed mechanism of tumor hyperprogression following COVID-19 vaccination. (**A**) Conceptual model illustrating how inoculation with mRNA vaccine leads to immune reactions depending on its biodistribution. Strong immunostimulation can override immunosurveillance of latent cancer cells and trigger tumor hyperprogression. (**B**) Schematic representation of the major immune cell types influencing tumor growth and immune regulation following mRNA vaccine exposure. LNP–encapsulated modified mRNA (modRNA/mRNA) interacts with innate immune sensors altering cytokine signaling (TNF-α, IL-1β, IL-6) and immune-cell polarization leading to immunosuppression and reduced cytotoxic CD8^+^ T-cell activity. Expansion of myeloid suppressor populations, along with pro-tumor cytokine feedback loops, fosters accelerated tumor cell proliferation and immune evasion. The imbalance between anti-tumor (M1, CD8^+^, NK) and pro-tumor (M2, Treg, MDSC) networks favors tumor hyperprogression.

The rapid appearance of cancer, the anatomical proximity of the tumors to vaccine sites, and the histologic signatures of inflammation the support immune mechanisms that promote the progression of latent clones rather than de novo carcinogenesis. We hypothesize two interrelated processes: localized inflammation and modulation of the tumor microenvironment with transient functional immunosuppression that relaxes immune surveillance. Might account for hyperprogression of latent or occult cancer cells (Figure 5).

Numerous studies (both human and animal) have shown that COVID mRNA vaccines and infection trigger production of proinflammatory cytokines including interleukin-6 (IL-6), TNF-α, and IL-1β within 1-3 days after vaccination [121–124]. In the case of vaccination, the reaction is due to the innate immune response to the mRNA and lipid nanoparticle (LNP) components, which activate pattern-recognition receptors TLR7/8 and NLRP3 [125–128]. Therefore, local production of these cytokines will occur wherever the mRNA and LNPs are biodistributed, which include the injection site, draining lymph nodes, as well as other distant sites [129].

IL6 activates STAT3 which drives cancer cell proliferation, survival, angiogenesis, and immune suppression in the tumor microenvironment [130]. TNF-α activates NF-κB and AP-1 that also drives cell survival, proliferation, angiogenesis, and immune evasion. TNF-α can create a self-sustaining inflammatory loop by recruiting myeloid-derived suppressor cells (MDSCs), tumor-associated macrophages (TAMs), and regulatory T cells (Tregs) that suppress cytotoxic T-cell activity and produce additional TNF-α, IL-6, and IL-10. TNF-α induces further expression of IL-6, CXCL1/2/8, and COX-2, which fosters further proliferation, angiogenesis, and immune evasion [130]. IL-1β upregulates VEGF, MMPs, and integrins, promoting neovascularization and extracellular matrix remodeling. IL-1β drives polarization of macrophages toward the M2-like (tumor-promoting) phenotype, expands Th17 cells and an neutrophils, thereby contributing to chronic inflammation and immunosuppressive tumor microenvironment [130].

Together IL-6, TNF-α, and IL-1β constitute a synergistic pro-inflammatory circuit capable of stimulating proliferation and angiogenesis. Together, this circuit is known to rapidly promote the development of cancer if transformed or pre-malignant cells already exist. Indeed, there are several reports demonstrating acceleration of pre-existing disease or reawakening from dormancy following inflammation [74, 131–134]. A synchronized surge of these three cytokines could provide a coordinated inflammatory storm that converts indolent or dormant transformed cells into rapidly proliferating, and angiogenic, malignancies (Figure 5). Therefore, research is needed to better understand whether the hypothesis that an inflammatory cytokine cascade is unleashed by mRNA vaccination could contribute to or even lead to post-vaccination cancer events.

In addition to influencing the behavior of pre-existing neoplastic cells, this transient cytokine-driven inflammatory surge could also modulate antiviral or antitumor immune surveillance. IL-6, TNF-α, and IL-1β recruit immunosuppressive myeloid populations and expand regulatory T cells, while dampening cytotoxic T-cell activity, functions that are essential for maintaining control of latent oncogenic viruses such as EBV, HHV-8, and MCV [135–137]. Accordingly, the observation that several post-vaccination cases involved virus-associated cancers (EBV, HHV-8, MCV) raises the possibility that short-lived alterations in immune surveillance may permit episodic viral reactivation or progression of virus-driven tumors. Mechanistically, transient impairment of cytotoxic T-cell surveillance prevents reactivation and replication of latent oncogenic viruses, leading to expression of viral oncogenes and proliferation of infected host cells. Similar processes are well documented in states of clinical immunosuppression, or hyperprogression in patients receiving immune checkpoint inhibitors [138–140].

Beyond innate cytokine induction, several studies have described transient adaptive immune changes following mRNA vaccination or acute infection [110, 141–145]. Although these findings are generally interpreted as reflecting physiological immunoregulation rather than overt immune dysfunction, they may correspond to short periods of reduced immune responsiveness [146–149]. If such windows occur, whether systemically or localized to specific tissue niches, they could, in theory, allow transient expansion of latent viral or neoplastic clones. At present, whether transient functional immunosuppression occurs after mRNA vaccination, and whether it is localized or systemic, remains unclear. Direct studies are needed to determine whether innate cytokine induction and adaptive immune modulation could contribute to or influence post-vaccination cancer events in susceptible individuals.

Evidence supporting these mechanisms have been suggested after SARS-CoV-2 infection itself. For example, Gregory et al. [74] reported aggressive glioblastoma at a median of 35 days following COVID-19 infection, an in one patient documented COVID-19 vaccine prior to diagnosis. The rapid onset of glioblastoma was attributed to immune disruption which may be in part due to the neurotropism of SARS-CoV-2 [150, 151] or the biodistribution of the LNPs [125, 127, 129] and the immune response to this. Indeed, Spike protein as has been localized to brain tissue as well as glioblastoma cells and macrophages surrounding the tumor cells [87]. Hu et al. [72] similarly demonstrated that direct viral exposure, and the presence of Spike protein, with cytokine-mediated injury in glioma organoids enhanced tumor cell proliferation and invasiveness, supporting a model of infection-driven tumor stimulation. For breast cancer, Chia et al. [74] showed that respiratory viral infections including SARS-CoV-2, could rapidly awaken dormant metastatic breast cancer cells in the lung through interferon-driven activation of the STAT1–NF-κB axis, which remodels the local niche into a pro-metastatic state. Both the virus and the mRNA vaccines engage innate immune sensors and elicit complex cytokine and interferon responses that can remodel the tumor–immune interface.

Together, these observations suggest that acute inflammatory activation, short-lived immune refractoriness, and transient lapses in cytotoxic surveillance form a biologically plausible framework through which vaccination could influence and promote the behavior of pre-existing or latent neoplastic cells. The immunological mechanism suggests that infection and vaccination may operate along a common biological continuum, differing primarily in intensity, biodistribution, and persistence of the immune and molecular perturbations they induce. Although direct causal evidence is not yet available, the convergence of innate cytokine surges, transient modulation of T- and B-cell dynamics, and signals associated with immune regulation highlights the importance of further study. Clarifying the magnitude, duration, and tissue specificity of these post-vaccination immune states will be essential for determining whether, and in which individuals, they have clinical relevance for cancer progression or recurrence.

## Spike protein biology

The transformation of a normal cell into a cancer cell involves disruption of multiple safeguards controlling cell growth, survival, and DNA repair. Laboratory studies have reported that the spike protein, whether it is produced by infection or by vaccination, has biological activities [110, 145, 152–158] with oncogenic potential [159–161]. For example, in addition to interacting with ACE2 receptors, spike protein fragments have been shown to interact with NRP-1, integrins, and TLRs leading to VEGF/NRP-1 signaling [155, 162, 163]. Spike protein has also been reported to induce DNA damage [160, 164, 165] and modify p53 pathway under oxidative stress [164, 166]. Therefore, in theory, such interactions of spike protein with these pathways could contribute to cellular transformation, both from the vaccine but also from infection, especially if the spike protein remains present long after vaccination or from multiple COVID infections.

The spike protein produced by the vaccines has been reported to persist for weeks, months and even years [109, 110, 116, 167–170] after vaccination, providing potential long-term activity in cells. Moreover, the stabilized Spike protein produced by the covid vaccines (Spike-2P), differs from the natural protein in SARS-CoV-2 as it contains two proline substitutions (K986P and V987P) that enable stabilization [171]. Because of this, it will be important to assess whether cancer incidence correlates with the variant spike protein expression (or persistence) in the body but also whether it is this version that is present in tumors as has been reported in glioma and astrocytoma [73] as well as metastatic breast cancer [36]. In a recent case report of metastatic breast cancer to the skin, the lesion appeared within one month after the 6th dose of vaccination (Pfizer-BioNTech) and the metastatic cancer cells stained for spike protein, but not for nucleocapsid protein of SARS-Cov-2 virus ([36], Figure 3F) suggesting it was vaccine-derived spike protein. Hence, chronic exposure to an agent with biological activity that disrupts cell cycle and DNA damage response pathways could represent a novel etiological factor to cancer. Of note and relevance to glioblastoma or other central nervous system (CNS) pathologies after either COVID infection or vaccination could be the CNS-tropism of spike protein [150, 151, 172].

## DNA contaminants

Residual DNA in biologics is a well-established and acknowledged byproduct of vaccine manufacturing, with limits set forth by the FDA and World Health Organization (WHO), but only for naked DNA, not LNP encapsulated DNA [173]. The DNA impurities in mRNA vaccines arise due to the byproduct of in-vitro transcription [174], and can include double strand DNA (dsDNA), abortive RNAs and RNA:DNA hybrids [103, 174]. They are encapsulated by nanolipids allowing for more stable and efficient access into cells increasing the risk of integration [128, 175, 176]. Furthermore, the residual DNA in the mRNA vaccine formulations [103–108] from the manufacturing process exceed the established limits even for naked DNA. Studies have directly compared the transfection efficiency of naked DNA to LNP encapsulated DNA and shown that integration of lipid-based transfection is significantly higher than naked DNA [175]. Moreover, skeletal and cardiac muscles are well known to take up (and even express naked plasmid DNA) *in vivo* [177–179]. Notably, a study of cardiac tumors in the post-COVID period revealed both a 1.5% increase in tumor incidence and the expression of spike protein with the tumors, particularly in those associated with vaccination [86].

The quantity of residual DNA reported in several independent assessments exceeds recognized limits for naked DNA, and the size distribution of DNA fragments, when combined with enhanced transfection efficiency due to LNPs raises the possibility of genomic insertion. In addition, because SV40 regulatory elements are present in the BNT2b vaccine impurities [180], when inserted into genome this DNA can alter the expression of adjacent sequences and/or normal gene regulation and increases tumorigenic potential [120, 181]. Foreign DNA, especially when delivered in the highly inflammatory LNPs [182] can activate innate immune sensing pathways, including the cytosolic cGAS–STING and endosomal Toll-like receptor 9 (TLR9), leading to type I interferon and inflammatory cytokine responses [183, 184].

The limits on DNA impurities were established for naked DNA [185], not LNP-encapsulated DNA which causes enhanced cellular uptake and intracellular persistence of DNA fragments. This will increase the opportunity for insertional mutagenesis leading to possible genomic rearrangements, as well as integration and expression of persistent spike protein, disruption of normal gene regulation, as well as possible activation of proto-oncogenic pathways, or inactivation of tumor suppressors. In fact, *in vitro* studies demonstrate genomic integration rates of ~1–10% of initially transfected cells with lipid-based delivery systems [120]. No studies have been conducted demonstrating that the level of DNA impurities present in the vaccines are insufficient to transfect cells, nor have studies ruled out the possibility of integration.

## Gaps in knowledge

Despite the unprecedented global scale of COVID-19 vaccination, profound gaps remain in our understanding of how mRNA vaccine platforms interact with fundamental pathways of host defense, tissue homeostasis, and tumor biology. These gaps span molecular, cellular, organismal, and population level biology. There is an absence of data linking mRNA vaccines, and especially COVID-19 mRNA vaccines to downstream biological consequences.

At the molecular level, major knowledge gaps concerning how chemical and structural modifications of the SARS-CoV-2 spike protein, nucleoside and amino acid substitutions (e.g., N^1^-methylpseudouridine), and LNP formulations influence host-cell signaling, genomic stability, and immune regulation. Vaccine engineering has focused on maximizing antiviral immunogenicity, yet far less is known about potential collateral interactions between spike protein expression, tumor-suppressive, DNA damage or stress response pathways that could be inadvertently modulated during intense immune activation or altered cellular signaling that impacts on host defenses against cancer.

The distinction between vaccination associated tumor initiation and promotion also remains unresolved. There is no empirical validation that vaccination only accelerates pre-existing disease rather than also initiating new malignancies. Because somatic mutations and dormant neoplastic cells are ubiquitous in adult tissues, short-latency tumor emergence over weeks to months may reflect the promotion of latent clones rather than de novo carcinogenesis, a phenomenon consistent with hyperprogression observed in subsets of patients receiving immune checkpoint inhibitors [138, 139]. Oncogenic drivers such as MDM2/MDM4 amplification or EGFR amplification, overexpression, or mutations have been implicated in hyperprogression [138, 140, 186] and metastatic aggressiveness [187, 188], suggesting that vaccine-induced cytokine or checkpoint shifts could theoretically converge on similar oncogenic pathways. Also unclear is how mRNA vaccines targeting SARS-CoV-2 might be sensitizing tumors to immune checkpoint inhibitors as has been recently suggested [19].

### Cellular and immunologic gaps

At the cellular level, there is limited mechanistic understanding of how mRNA vaccine components, spike protein persistence, or repeated immune activation shape innate–adaptive immune crosstalk, particularly in dendritic cells, macrophages, and stromal compartments. The molecular triggers and long-term immune consequences of a hyperinflammatory response observed in both severe COVID-19 and rare post-vaccination events [189] remain poorly characterized. Equally unexplored is the role of the microbiome in modulating vaccine responsiveness, systemic inflammation, and tumor promotion. Understanding how antigen persistence, cytokine polarization, and pattern-recognition receptor activation influence local tissue remodeling, cellular senescence, and pro-tumorigenic inflammation represents a critical unmet need.

### Host susceptibility and biodistribution

At the organismal level, the greatest uncertainty lies in the heterogeneity of host susceptibility. For example, the heterogeneity of individual differences in baseline state of activation, responsiveness, and regulation in response to COVID-19 vaccination has been reported [190]. Furthermore, heterogeneity of DNA repair, epigenetic plasticity, and cytokine response level is also not well understood and likely modulates vaccine response and risk. Variable LNP biodistribution, including uptake in liver, spleen, bone marrow, and lymphoid tissues, may alter both immune potency and potential off-target effects, yet these parameters have not been systematically profiled in humans. There is also a lack of data on how vaccination during or shortly after SARS-CoV-2 infection, or cumulative exposure to multiple mRNA doses, affects long-term immune homeostasis and tumor surveillance. Interactions between vaccine-induced inflammation and latent oncogenic viruses (e.g., EBV, HHV-8, MCPyV) remain particularly underexplored.

### Population and epidemiologic gaps

At the population level, large-scale epidemiologic studies remain limited and often inconclusive. Existing registries rarely integrate molecular or immunologic correlates, hindering causal inference. Moreover, current pharmacovigilance systems were not designed to detect rare but biologically informative oncologic events, creating a blind spot between individual case reporting and aggregated safety analyses. Robust longitudinal follow-up of vaccinated cohorts with integrated molecular profiling will be essential to distinguish true biological signals from background incidence and to identify susceptible subgroups.

### Alternative preventive and therapeutic strategies

Addressing key knowledge gaps will require exploration of complementary antiviral strategies that reduce infection risk while minimizing host perturbation. For example, MEK inhibitors have been shown to suppress ACE2 expression and viral entry [191], yet host-directed antivirals have received little attention. Likewise, enhancing innate immunity through agents that boost pattern-recognition receptor signaling or interferon responses could theoretically mitigate both COVID-19 infection and cancer risk by restoring balanced immune surveillance. Indeed, there are approaches validated by FDA of approved immunomodulators [192] and emerging analogs [193].

Major gaps exist in forensic evidence to evaluate the spread of COVID infection or COVID vaccine Spike protein into cancer tissues as well as effects on cellular growth control pathways *in vivo*. We currently do not have tissue-level, mechanistic, or molecular studies that trace where spike protein localizes in the body after infection or vaccination, especially in tumors. No systematic research has mapped spike protein distribution within tumors (solid or hematologic), either through immunohistochemistry, mass spectrometry, RNA *in situ* hybridization, or other molecular tissue-tracking methods. There have been essentially no in-vivo studies (animal or human tumor samples) showing how spike protein exposure affects proliferation, signaling, apoptosis pathways, tumor–immune interactions, or oncogene/tumor suppressor regulation.

In sum, the field faces interlocking knowledge gaps encompassing incomplete molecular characterization of vaccine–host interactions, insufficient mechanistic study of immune dysregulation and tissue remodeling, poorly defined host susceptibility factors, and limited longitudinal surveillance. Bridging these gaps will require multi-scale, interdisciplinary research.

## Limitations

The findings of this review should be interpreted within several important limitations inherent to the available literature and study design. First, although there are larger studies published, most reports are single-patient case descriptions or small case series. Many reports lack documentation of pre-existing conditions, prior oncologic history, concurrent infections, or medications that could confound interpretation. Therefore, while these observations are valuable for early signal detection, they are highly susceptible to publication bias and selective reporting. It is plausible that cases perceived as unusual or temporally linked to vaccination are preferentially submitted for publication, while the absence of comparable control observations limits inference regarding incidence or relative risk.

Second, the heterogeneity of the studies that span both mRNA and non-mRNA vaccine platforms, doses, populations, and diagnostic standardization introduces important variabilities when it comes to the associations between mRNA vaccines and other COVID-19 vaccines. The mechanistic hypotheses proposed here remain speculative in the absence of direct *in vivo* validation. No current studies have demonstrated oncogenic transformation or tumor initiation causally attributable to the COVID mRNA vaccine or its components. Nor have animal studies demonstrated vaccine-induced tumor promotion either. These mechanisms should therefore be regarded as biologically plausible models that warrant targeted experimental study.

Finally, this literature review relied on publicly available literature that lacked standard medical subject headings (MeSH) indexing for peer-reviewed papers. The results of this review demonstrate that there indeed exists a body of literature on this subject matter across many journals that not well indexed or cross-referenced using obvious indexing vocabularies that prevent them from being retrieved by conventional database queries. This imposes inherent limitations on both data completeness and verification but also in identifying knowledge gaps.

## Ethical considerations

Different disciplines adopt different standards for evidence used to determine standard of care that should be shared widely. In 2025, there is a major divide about published evidence that doesn’t reach a certain standard at a population level being viewed by some as “misinformation,” or “fearmongering.” In the field of Oncology, there is a standard that has been in place for decades regarding how adverse events are viewed in the process of drug development as well as in clinical practice. A single serious adverse event is reportable to institutional review boards (IRB’s) and regulatory agencies such as the FDA. The mRNA vaccines incorporate mechanisms commonly associated with gene-therapy technologies, though their regulatory review followed the vaccine pathway rather than the criteria normally applied to human gene-therapy products, which rigorously evaluate. Cancer (including insertional mutagenesis, clonal expansion, leukemogenesis, and treatment-related malignancy) is a key safety endpoint that gene-therapy regulations are explicitly designed to monitor, but vaccines are not.

Regarding COVID mRNA vaccines, in the US and globally, there is no informed consent that captures all known and potential adverse events. All known and potentially serious adverse events are required to be disclosed in vaccine information sheets, informed consent forms, and it is important for physicians and other health care providers to be well-educated concerning risks regardless of how rare they are. An important example that led to practice change in the US after decades which lacked DPYD testing. This now mandated test identifies pathogenic or reduced-function variants in the DPYD (dihydropyrimidine dehydrogenase) gene that impairs 5-FU/capecitabine metabolism. Changes in practice occurred recently and has become in line with European practice to reduce the risk of severe toxicity from exposure to 5-fluorouracil chemotherapy.

Cancer susceptibility varies among individuals in the population as governed by genetic and environmental factors and a growing body of literature adds socioeconomic factors that interact with the other two factors in complex ways. It is likely that the susceptibility to cancer following COVID vaccines varies greatly within the population with some individuals at greater risk. It is important to recognize these issues and to study them in order to develop improved guidance for a risk: benefit analysis in the setting of informed consent.

The long timeline required to establish or exclude causality in oncology is often measured in years or decades. This should not lessen the immediate ethical responsibility to provide accurate and current information to individuals considering vaccination or additional boosters. The fact that definitive causal inference demands mechanistic studies, longitudinal cohorts, and large epidemiologic analyses should not be taken as a rationale for withholding emerging clinical observations or biologically plausible concerns. Ensuring that clinicians and patients have access to evolving evidence, including rare but mechanistically credible adverse events, is consistent with the core principles of medical ethics and reinforces public trust by clearly delineating what is known, what remains unresolved, and what work is ongoing. Informed consent must adapt as new data accumulate, even when causal questions remain open, and doing so does not assume bias to what future investigation will ultimately show.

## CONCLUSIONS

The collective world-wide evidence from 2020–2025 underscores a biologically plausible connection between COVID-19 vaccination and cancer. The recurring clinical findings documented across many reports of de-novo cancer onset, rapid tumor progression, viral reactivation, and reawakening of dormant disease, highlight critical gaps in knowledge and understanding of how large-scale immune changes produced by the vaccine interacts with cancer biology.

Both SARS-CoV-2 infection and COVID-19 vaccination engage overlapping biological pathways that could, in principle, influence cancer risk, yet they differ in mechanism, magnitude, biodistribution, and duration of their effects. Shared mechanisms include activation of the innate immune system, robust interferon signaling, cytokine induction, oxidative stress, and transient disruption of immune-cell homeostasis. These changes can theoretically expose latent malignancies, promote clonal expansion of preexisting mutant cells, or create microenvironmental contexts permissive to tumor progression.

In addition, both infection and vaccination induce spike protein expression, which interacts with ACE2-expressing tissues and can trigger endothelial activation, inflammation, and cellular stress pathways implicated in oncogenic signaling. Both can also lead to prolonged inflammatory and tissue-injury states, all of which could contribute to genomic instability, epigenetic remodeling, and chronic immune dysregulation.

However, unique mechanisms distinguish COVID-19 vaccination from natural infection. Vaccination involves widespread biodistribution, intracellular uptake and persistence of modified nucleic acid templates that drive synthesis of an unnatural Spike protein both at the injection site but also throughout the body. The presence of the residual or fragmented DNA combined with the LNP-mediated delivery to immune and non-immune tissues, and sustained spike expression for month to years represent vaccine-specific factors that could theoretically promote insertional mutagenesis, perturb immune surveillance, or accelerate growth of preexisting malignant clones. As such there is much to be learned from human tissue and blood samples as well as autopsies to better understand the interplay between COVID infection, vaccination and cancer mechanisms.

To this end, Spike protein presence and persistence along with the biological effects that are cell autonomous or that depend on host immune interactions need to be studied to establish connections to cancer initiation and progression. Accordingly, we propose that tumors arising after documented SARS-CoV-2 infection or following COVID-19 vaccination be evaluated using a standardized immunohistochemical classification framework.

At minimum, this should include assessment of viral antigen expression patterns by IHC. Spike-positive/nucleocapsid-positive, spike-positive/nucleocapsid-negative, and spike-negative/nucleocapsid-negative phenotypes should be defined. This assessment should be integrated with detailed characterization of proliferative activity (e.g., Ki-67), cell-death and DNA-damage response markers, tumor-suppressor and oncogene pathway signatures, and the immune tumor microenvironment.

Implementing this type of reporting across clinical pathology and autopsy evaluations would allow more precise discrimination among tumors potentially driven by infection, by vaccine-related antigen expression, or by unrelated oncogenic processes, and would enable aggregation of comparable cases across institutions. Establishing uniform criteria of this kind is essential for building a coherent evidence base, supporting mechanistic research, and ultimately determining whether observed associations reflect coincidence, unmasking of latent disease, immune perturbation, or true causal relationships.

Establishing causality between SARS-CoV-2 infection, COVID-19 vaccination, and cancer requires a level of evidence far beyond temporal association. In oncology, causation is never determined by a single observation or study; it emerges only when multiple, independent lines of evidence converge over time. This includes mechanistic data (such as genomic integration analyses, clonal evolution trajectories, immune-profiling, and epigenetic changes), pathology-based findings (including autopsies with molecular characterization), experimental models that accurately reflect human tissue biology (organoids, humanized systems, long-read sequencing of exposed tissues), and population-level epidemiologic studies capable of detecting small but meaningful signals against background incidence. Only by integrating these approaches can we distinguish coincidence from unmasking of latent disease, expansion of preexisting malignant clones, or true de novo oncogenesis. Importantly, the need for rigorous evidence should not be used to dismiss emerging patterns.

Transparent discussion of biologically plausible mechanisms and surveillance strategies is essential to determine if this temporal association is causally linked. Current reliable epidemiologic data is lacking to provide evidence that vaccination does not increase population-level cancer incidence. Peer-reviewed literature is not completely or easily indexed. Establishing a framework for post-vaccination cancer surveillance, could help detect rare adverse patterns early and enable mechanistic follow-up without compromising public confidence. The goal of this review is not to estimate population-level cancer risk but to provide a structured synthesis of the existing peer-reviewed literature, identify recurring clinical and biological themes, and delineate critical gaps that require rigorous epidemiologic and mechanistic follow-up. This will enable a better understanding of the full spectrum of immune responses to inform safer immunization strategies and illuminate previously underappreciated links between immunity and cancer biology.

The scientific imperative moving forward should be a coordinated framework combining longitudinal surveillance and mechanistic experimentation to allow us to distinguish coincidence from causality and to refine future vaccine platforms accordingly. In doing so, we stand to gain not only a clearer understanding of vaccine safety, but also a deeper insight into the fundamental links between immunity, infection, and cancer emergence.

## SUPPLEMENTARY MATERIALS


