# Supplementary material for: COVID vaccination and post-infection cancer signals: Evaluating patterns and potential biological mechanisms
Source: Oncotarget. 2026 Jan 3;17:1–29. doi: 10.18632/oncotarget.28824 (PMC12893478; doi:10.18632/oncotarget.28824)
Supplement: Supplementary file 1 [file oncotarget-17-28824-s001.pdf]

COVID vaccination and post-infection cancer signals: Evaluating patterns and potential biological mechanisms

SUPPLEMENTARY MATERIALS

Supplementary Table 1: Summary of figure permissions and allowed use

References	Figure	Permission	License/ Permission basis	Type of Use Permitted	Journal/Date/ Publisher
Abue et al.	Figure 3E	Explicit author permission	Open access; permission confirmed by authors	Reproduction in review article	*Cancers 2025* © MDPI.
Bae et al.	Figure 3C	Explicit author permission	Open access; permission confirmed by authors	Reproduction in review article	*Cureus 2023* © Springer Nature
Kim et al.	Figure 3J	Explicit publisher permission	Open access; permission confirmed by publisher	Reprinted with permission in review article	*Clin Endosc. 2024 57(3)402-406)* © Korean Society of Gastrointestinal Endoscopy
Gullotti et al.	Figure 3H	Publisher license purchased	Publisher-issued reuse license	Reproduction in review article	*Radiol Case Rep*. 2022 © Elsevier.
Sano et al.	Figure 3F	Publisher license purchased	Publisher-issued reuse license	Reproduction in review article	*J. Derm Sci*, 2025. © Elsevier.
O'Sullivan et al.	Figure 3I	Publisher license purchased	Publisher-issued reuse license	Reproduction in review article	*J of Neurology*. 2021 © Elsevier
Wagle et al.	Figure 3G	Publisher license purchased	Publisher-issued reuse license	Reproduction in review article	*Indian J Ophthalmo* 2022 © Wolters Kluwer
Cavanna et al.	Table 3, Figure 3A	Open access (no email permission required)	MDPI CC BY license	Reproduction and/ or adaptation with attribution	*Medicina, 2023.* © MDPI.
Li et al.	Figure 3D	Open access (no email permission required)	MDPI CC BY license	Reproduction and/ or adaptation with attribution	*Front Med*, 2022. © Frontiers
Sekizawa et al.	Figure 3B	Open access (no email permission required)	Creative Commons CC BY 4.0	Unrestricted reuse with attribution	*Front Med*, 2022. © Frontiers
Sasa et al.	Figure 3K	Open access (no email permission required)	Springer Nature CC BY license	Unrestricted reuse with attribution	*Surg Case Rep* 2022 © Springer Nature.

All reproduced figures and tables are used in accordance with publisher licenses, Creative Commons terms, or explicit author permission. Where applicable, figures are reproduced or adapted with attribution as required by CC BY licensing.
